# Pathogenic Germline Variants in Uveal Melanoma Driver and BAP1‐Associated Genes in Finnish Patients with Uveal Melanoma

**DOI:** 10.1111/pcmr.13198

**Published:** 2024-09-30

**Authors:** Pauliina E. Repo, Eveliina Jakkula, Juho Hiltunen, Heidi Putkuri, Aleksandra Staskiewicz‐Tuikkanen, Reetta‐Stiina Järvinen, Martin Täll, Virpi Raivio, Rana'a T. Al‐Jamal, Tero T. Kivelä, Joni A. Turunen

**Affiliations:** ^1^ Eye Genetics Group, Folkhälsan Research Center, Biomedicum Helsinki Helsinki Finland; ^2^ Ocular Oncology Service, Department of Ophthalmology University of Helsinki and Helsinki University Hospital Helsinki Finland; ^3^ Department of Clinical Genetics University of Helsinki and Helsinki University Hospital Helsinki Finland; ^4^ Ophthalmic Genetics and Rare eye Diseases Service, Department of Ophthalmology University of Helsinki and Helsinki University Hospital Helsinki Finland

**Keywords:** BAP1, cancer syndrome, genetic predisposition, uveal melanoma

## Abstract

Uveal melanoma (UM) is a rare yet aggressive eye cancer causing over 50% mortality from metastasis. Familial UM, amounting to 1%–6% of patients in Finland and the United States, mostly lack identified genetic cause, while 8% show associations with other cancer syndromes. We searched novel genetic associations for predisposition to UM, additional to already studied *BAP1* and *MBD4*, by using targeted amplicon sequencing of 19 genes associated with UM, BAP1, or renal cell carcinoma in 270 consecutively enrolled Finnish patients with UM. Key UM drivers *GNAQ*, *GNA11*, *CYSLTR2*, *PLCB4*, *EIF1AX*, and *SF3B1* lacked pathogenic germline variants. One patient carried the pathogenic *BRCA1* variant c.3626del p.(Leu1209*), and one harbored a novel truncating *MET* variant c.252C > G p.(Tyr84*), classified as likely pathogenic. *FLCN* and *BRCA2*, previously identified with pathogenic variants in patients with UM, did not have such variants in our cohort. Two patients were heterozygous for a pathogenic recessive *BLM* variant c.2824‐2A > T. None of the carriers of identified variants had familial UM. We identified *BRCA1* and *MET* as genes with pathogenic germline variants in Finnish UM patients, each with a frequency of 0.4% (95% confidence interval, 0–2).

## Graphical abstract

We enrolled a consecutive cohort of 270 Finnish patients with uveal melanoma (UM) and analyzed them for pathogenic germline variants in 19 genes associated with UM, BAP1, or renal cell carcinoma (RCC). One patient carried the pathogenic *BRCA1* variant c.3626del; p.(Leu1209*), while another had a likely pathogenic *MET* variant c.252C>G; p.(Tyr84*). Additionally, two patients were identified as heterozygous for the recessive pathogenic *BLM* variant c.2824‐2A>T.

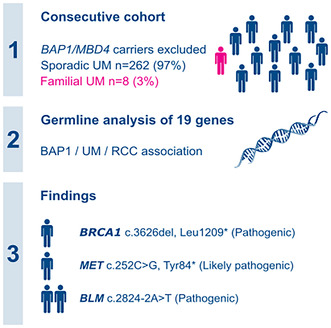

**Summary**

- Taken together with our previous studies, a broader germline analysis of 270 consecutively enrolled Finnish patients with uveal melanoma (UM) reaffirms *BAP1* as the primary contributor to genetic predisposition to UM.
- No pathogenic germline variants in other UM drivers were identified.
- Among previously reported genes, a pathogenic *BRCA1* variant was identified in one patient in our cohort, whereas no such variants were found in *BRCA2* or *FLCN*.
- A likely pathogenic *MET* variant warrants further investigation. No pathogenic germline variants were found in eight familial UM patients, highlighting need for continued research.

Uveal melanoma (UM), the most prevalent primary intraocular cancer in adults, originates from melanocytes of the choroid, ciliary body, or iris, typically because of one of a few well‐established somatic driver events. Approximately 90% of tumors exhibit mutually exclusive mutations in the G‐protein pathway genes *GNAQ* or *GNA11*, while 4%–5% harbor mutations in their downstream effector *PLCB4*, and 2% in *CYSLTR2* coding for an upstream G‐protein‐coupled receptor (Field et al. 2018; Johansson et al. 2016; Moore et al. 2016; Robertson et al. 2017; Shain et al. 2019; Van Raamsdonk et al. 2009, 2010). Subsequent mutations, also typically mutually exclusive, occur in *BAP1*, *EIF1AX*, and *SF3B1* in 50%, 14%–22% and 11%–19% of tumors, respectively, correlating with high, low, and intermediate risk of metastasis (Field et al. 2018; Johansson et al. 2020; Robertson et al. 2017; Rodrigues et al. 2019; Royer‐Bertrand et al. 2016; Shain et al. 2019). *MBD4* loss is observed in up to 4% of primary UM in France, contributing to a distinct hypermutated tumor phenotype (Derrien et al. 2021; Johansson et al. 2019; Rodrigues et al. 2018; Saint‐Ghislain et al. 2022).

*MBD4* and, especially, *BAP1* are also associated with inherited UM predisposition. Pathogenic germline *BAP1* variants are identified in ~2% of all patients and in 20%–25% of those with familial UM (Gupta et al. 2015; Pilarski et al. 1993; Rai et al. 2017; Repo et al. 2019; Turunen et al. 2016). UM is one core tumor in dominantly inherited BAP1 tumor predisposition syndrome (BAP1‐TPDS, OMIM 614327), along with mesothelioma, cutaneous melanoma, and clear cell renal cell carcinoma (Carbone et al. 2022). Deleterious germline *MBD4* variants cause recessive tumor predisposition syndrome 2 (TPDS2; OMIM 619975), linked especially to acute myeloid leukemia, colorectal cancer, and UM (Palles et al. 2022). Heterozygous carriers of these variants are estimated to have a 9.15‐fold higher incidence of UM compared to the general French population (Derrien et al. 2021). Additionally, patients with UM have been identified with pathogenic germline variants in 15 other cancer‐associated genes (Abdel‐Rahman et al. 2020; Cruz et al. 2011; Fontcuberta et al. 2011; Hajkova et al. 2018; Hearle et al. 2003; Huang et al. 2018; Iscovich et al. 2002; Kannengiesser et al. 2003; Lobo et al. 2017; Nathan et al. 2021; Sinilnikova et al. 1999).

Pathogenic variants in UM tumorigenesis and BAP1‐associated genes result in diverse outcomes depending on whether they are somatic, inherited, or *de novo*, with the extent of possible mosaicism further shaping the phenotype. *De novo* missense variants in *BAP1* are associated with neurodevelopmental Kury–Isidor syndrome, with assumed cancer predisposition similar to BAP1‐TPDS (Kury et al. 2022). Kury–Isidor syndrome shares features with Bohring–Opitz, Shashi–Pena and Bainbridg–eRopers syndromes, caused by mosaic or *de novo* pathogenic variants in *ASXL1*/*2*/*3* genes, with unclear cancer predisposition (Cuddapah et al. 2021). ASXL proteins interact with BAP1 and are somatically altered particularly in hematological cancers (Micol and Abdel‐Wahab 2016). Mosaicism for pathogenic *GNAQ* variants, and rarely for *GNA11*, causes vascular abnormalities ranging from port‐wine birthmarks to Sturge–Weber syndrome, with glaucoma and neurological involvement that depend on mosaicism extent (Dompmartin et al. 2022; Van Trigt, Kelly, and Hughes 2022). Germline pathogenic variants in *GNA11* are also associated with dominantly inherited hypo‐ and hypercalcemia (Howles et al. 2023), while those in *PLCB4* with dominantly and recessively inherited auriculocondylar syndrome (Nabil et al. 2020). To our knowledge, no cancer phenotype is reported with *GNAQ* and *GNA11* associated Sturge–Weber syndrome, *GNA11* associated hypo‐ or hypercalcemia, or *PLCB4* associated auriculocondylar syndrome, although the function of these genes is somatically altered in uveal melanomas. *CYSLTR2*, *EIF1AX*, and *SF3B1*, have not been associated with inherited conditions so far, although the latter is frequently altered in myeloid malignancies in addition to UMs.

The genetic basis of familial UM (FUM) remains unresolved in 75%–80% of patients (Abdel‐Rahman et al. 2020; Rai et al. 2017; Repo et al. 2019). Consequently, genes other than *BAP1* and *MBD4* likely predispose to UM. An additional 8% might emerge in families with other cancer syndromes such as breast‐ovarian cancer syndrome, familial melanoma, or Lynch syndrome (Abdel‐Rahman et al. 2010). Further, renal cell carcinoma (RCC) has been hypothesized to have genetic similarities with UM (Singla 2020). To elucidate additional genetic factors that may predispose to UM, we conducted germline targeted amplicon sequencing of 19 genes in a consecutive cohort of patients with UM, originating from Finland, a country from Northern Europe, a region with a higher than average incidence of UM in Europe (Kivelä 2014; Virgili et al. 2007). We analyzed pathogenic germline variants within coding regions of six genes associated with UM tumorigenesis, seven linked to BAP1 functions, and seven confirmed or candidate genes for RCC, a cancer that in our service was the most common nonocular malignancy associated with BAP1‐TPDS, the co‐occurrence of which with UM we at the time also explored in patients outside the present, consecutive series of UM. The latter included three genes previously linked to UM in isolated patients, of which BRCA1 is known to interact with BAP1 (Figure S1).

We enrolled 270 (52%) of the 521 Finnish patients consecutively diagnosed with UM at the Ocular Oncology Service, Helsinki University Hospital, a national referral center, between 2010 and 2017, who were willing to participate and whose samples were available at the time of the sequencing. All patients had been previously analyzed for pathogenic germline variants in *BAP1* and *MBD4* (Repo et al. 2020, 2019; Turunen et al. 2016). Eight patients who were pathogenic variant carriers were excluded from the present study (8 with *BAP1*, none carried *MBD4*). The patients were queried for history of previous cancers at the time of diagnosis, and subsequently during annual follow‐up visits. Reported nonocular cancers were verified from patient records. Detailed patient characteristics of all eligible patients is reported in Repo et al. (2019) Table S1. The project was approved by the institutional review board of the Hospital Region of Helsinki and Uusimaa on June 11, 2012, and it followed the tenets of the Declaration of Helsinki.

Genomic DNA was extracted from peripheral whole blood using standard methods. The targeted genes were selected based on the literature (Johansson et al. 2016; Moore et al. 2016; Royer‐Bertrand et al. 2016; Van Raamsdonk et al. 2009; Van Raamsdonk et al. 2010), STRING (Szklarczyk et al. 2019) and the Cancer Gene Census databases (Sondka et al. 2018). The sequencing probes were designed with DesignStudio Sequencing Assay Designer (Illumina, San Diego, CA). Library preparation was done using TruSeq Custom Amplicon kit v1.5 (Illumina), and samples were sequenced with HiSeq 2500 (Illumina) at the Institute for Molecular Medicine Finland (FIMM, Helsinki, Finland). The read alignment to Genome Reference Consortium Human Build 37 (GRCh37) has been described previously (Repo et al. 2019). We included only variants with a read depth of at least 10 and a variant allele frequency (VAF) of 30% or higher. The remaining variants were annotated with Annotate Variation (ANNOVAR) (Wang, Li, and Hakonarson 2010). All variants reported as pathogenic or likely pathogenic in ClinVar were included in further analysis. Additionally, nonsynonymous exonic and splicing region (± 2 bp) variants with minor allele frequency in gnomAD v2.1.1 (Karczewski et al. 2020) < 0.001 were analyzed if they received deleterious MetaRNN (Li et al. 2022) prediction and were not reported as benign or likely benign. VarSome (Kopanos et al. 2019) was utilized to assign The American College of Medical Genetics and Genomics (ACMG) classification to the remaining variants (Richards et al. 2015). Pathogenic and likely pathogenic variants and variants of unknown significance (VUS) were visualized with Integrative Genomics Viewer (IGV) (Robinson et al. 2011) and looked up from gnomAD v.4.1.0. The Cancer Genome Atlas (TCGA) database at www.cancer.gov/tcga, and the Catalogue of Somatic Mutations in Cancer (COSMIC) at https://cancer.sanger.ac.uk/cosmic were utilized when relevant. Carrier frequencies with 95% confidence intervals (CI) were calculated using the modified Wald method. Two‐sided Fisher's exact test was using to probe association between carrier status and UM with RStudio 4.1.0.

The median age at diagnosis of UM was 64 years (range, 15–89; Table 1). Eight patients (3%) from seven families reported a relative with UM. One family included a pair of cousins, both enrolled, while the rest had one first‐degree relative (1 family), cousin (2 families) or a more distant relative (3 families) with UM. Two patients with FUM also had clear cell type RCC and one of them had cutaneous melanoma as third cancer. Additional to their UM, 14 patients have had prostate cancer, 11 breast cancer, and 6 RCC, and one patient a benign renal oncocytoma. In total, 53 (20%; 95% CI, 15–25) of the enrolled patients had a nonocular primary malignancy, seven of whom had 2 and one had 3 such cancers (Table 1).

**TABLE 1:** Characteristics of 270 eligible Finnish patients with primary uveal melanoma at the time of diagnosis and their disease outcome.

Characteristic	
Gender, *n* (%)	
Male	141 (52)
Female	129 (48)
Median age, years (range)	64 (15–89)
Age, years	
10–29	4 (1)
30–39	8 (3)
40–49	27 (10)
50–59	57 (21)
60–69	103 (38)
70–79	50 (19)
80–99	21 (8)
Younger than 45 years	
No	249 (92)
Yes	21 (8)
Family history of uveal melanoma, *n* (%)	
No	262 (97)
Yes	8 (3)
Median thickness, mm (range)	3.9 (0.2–19.4)
Median largest basal diameter, mm (range)	11.8 (1.2–26.0)
Location, *n* (%)	
Iris only	9 (3)
Ciliary body and choroid	56 (21)
Choroid only	205 (76)
Extraocular extension, *n* (%)	
No	261 (97)
Yes	9 (3)
TNM category, *n* (%)	
T1	98 (36)
T2	77 (29)
T3	67 (25)
T4	28 (10)
TNM stage, *n* (%)	
I	89 (33)
IIA	71 (26)
IIB	45 (17)
IIIA	38 (14)
IIIB	12 (4)
IIIC	3 (1)
IV	3 (1)
Not defined^a^	9 (3)
Number of nonocular cancers, *n* (%)	
None	216 (80)
One	46 (17)
Two	7 (3)
Three	1 (<1)
Type of nonocular cancer, *n* (%)	
Prostate carcinoma	14 (5/10)^b^
Breast carcinoma	12 (4/9)^c^
Renal cell carcinoma^d^	6 (2)
Thyroid carcinoma	5 (2)
Intestinal carcinoma^e^	5 (2)
Cutaneous melanoma	5 (2)
Lymphoma	4 (1)
Cutaneous basal cell carcinoma	3 (1)
Cutaneous squamous cell carcinoma	2 (<1)
Pancreatic adenocarcinoma	1 (<1)
Neuroendocrine small cell pulmonary carcinoma	1 (<1)
Uterine carcinoma	1 (<1/<1)^c^
Leukemia	1 (<1)
Sarcoma	1 (<1)
Adenocarcinoma, uncertain primary	1 (<1)
Metastatic uveal melanoma, *n* (%)	
No	201 (73)
Yes	73 (27)
Survival status, *n* (%)	
Alive	166 (61)
Dead of metastatic uveal melanoma	70 (26)
Dead of nonocular cancer	16 (6)
Dead of nonmalignant disease	18 (7)

Abbreviation: TNM, Tumor, Node, Metastasis classification, 8th edition.

^a^ Stage is not defined for iris melanoma.

^b^ All/male patients.

^c^ All/female patients.

^d^ Clear cell type (4), mixed clear cell and papillary type (1), and papillary type (1).

^e^ Adenocarcinoma of the colon (2), rectum (1), sigma (1), and appendix (1).

Dominant cancer‐associated pathogenic and likely pathogenic variants were identified in *BRCA1* and *MET*, and two patients were carriers of recessive pathogenic *BLM* variants. The pathogenic *BRCA1* variant c.3626del p.(Leu1209*), associated with dominant familial breast‐ovarian cancer (Sarantaus et al. 2000), was detected in a male diagnosed with a T2b, Stage IIB, UM at the age of 54, without family history of UM (Table 2, Figure S2). The overall allele frequency of the variant is 4.0 × 10^−6^ in gnomAD v.2.1.1, with no Finnish carriers, and 1.9 × 10^−6^ in gnomAD v.4.1.0, with a carrier frequency of 1 in 10,673 Finns (0.0094%, 95% CI 1 in 5,000 to 1 in 19). In the Finnish UM cohort, the carrier frequency is 1 in 270 (0.37%, 95% CI 0.009 to 2.05), and not significantly different from the frequency of probable loss‐of‐function (pLOF) *BRCA1* variant carriers observed in Finns in general (Table 3).

**TABLE 2:** Rare pathogenic and likely pathogenic variants and variants of unknown significance (VUS) in 270 Finnish patients with primary uveal melanoma (UM). Age refers to the age of the patient at the time of UM diagnosis.

Gene (transcript)	Variant	ACMG Classification	ClinVar variation ID	Gene Associated malignancy risk	Sex/age	TNM	Site	Height/LBD mm	Status Metastases	FUP years	Nonocular cancers	Familial UM
*BRCA1* (NM_007294.4)	c.3626del Leu1209* rs80357571	Pathogenic (PVS1, PP5, PM2)	54,943	AD Familial breast‐ovarian cancer (OMIM 604370), high risk AD Pancreatic adenocarcinoma (OMIM 614320), moderate risk AR Fanconi anemia, complementation group S (OMIM 617883), high risk other malignancies, risk/association pending confirmation	M/54	T2b Stage IIB	Choroid	5.4/12.7	Dead No	6 years 1 months	No	No
*MET* (NM_000245.4)	c.252C > G Tyr84* rs1584876265	Likely Pathogenic (PVS1, PM2)	—	AD Papillary renal cell carcinoma‐1 (OMIM 605074), high risk	M/54	T2a Stage IIA	Choroid	5.0/10.8	Alive No	10 years 2months	No	No
*BLM* (NM_000057.4)	c.2824‐2A > T rs745538883	Pathogenic (PVS1, PM2, PP5)	371,621	AR Bloom syndrome (OMIM 210900, risk of various cancers at young age) AD breast, mesothelioma, colorectal cancer, risk/association pending confirmation	M/45	T3a Stage IIB	Choroid	10.6/11.2	Alive No	8 years 10months	No	No
					M/64	T4b Stage IIIB	Choroid	12.0/18.2	Dead Yes	2 years 11months	No	No
	c.2489C > T Thr830Met rs759545027	VUS (PP3, PM2, BP1)	1,337,765		F/53	T1a Stage I	Choroid	2.4/7.3	Alive No	10 years 2months	Breast cancer age 62 years	No

Abbreviations: ACMG, The American College of Medical Genetics and Genomics; AD, autosomal dominant; AR, autosomal recessive; BP1, missense variant in a gene for which primarily truncating variants are known to cause disease; F, female; FUP, follow‐up time; LBD, largest basal diameter; M, male; PM2, Absent or very rare from race‐matched controls gnomAD; PP3, multiple lines of computational evidence support a deleterious effect; PP5, reputable source recently reports variant as pathogenic, but the evidence is not available to the laboratory to perform an independent evaluation; PVS1, Null variant in a gene where loss of function is a known mechanism of disease; TNM, tumor, node, metastasis classification.

**TABLE 3:** Frequency of pathogenic germline variant carriers in Finnish (FIN) patients with uveal melanoma (UM) compared to all probable loss‐of‐function (pLOF) variant carriers reported in gnomAD, by gene.

Gene	UM patients	pLOF gnomAD FIN*	*p* value (Two‐sided Fisher's exact test)	Odds ratio (95% CI)
*BRCA1*	1/270 (0.37%)	18/32,026 (0.056%)	*p* = 0.15	7 (95% CI 0–42)
*BLM*	2/270 (0.74%)	27/32,026 (0.084%)	p = 0.024	9 (95% CI 1–35)

Abbreviation: CI, confidence intervals.

* Documentation of gnomAD v.4.1.0 includes annotation of 32,026 Finnish individuals. No homozygous individuals for variants in either gene were represented in gnomAD v.4.1.0 FIN, and the number of carriers for all variants annotated as pLOF was extracted directly. Variants reported as benign, likely benign, or of uncertain significance (VUS) in ClinVar, or flagged with low confidence annotation, were excluded.

A truncating likely pathogenic *MET* variant c.252C > G, p.(Tyr84*) was identified in another male diagnosed with a T2a, Stage IIA, UM at the age of 54 (Table 2, Figure S2). Variants in the *MET* proto‐oncogene that are associated with RCC typically exhibit gain‐of‐function characteristics, resulting in increased MET protein levels or activity (Tovar and Graveel 2017). The patient had no history of any nonocular cancer, and no metastases were detected during follow‐up of over 10 years. This variant is neither present in the general population according to gnomAD nor in the TCGA or COSMIC databases that list somatic variants in cancers. *MET* c.252C > G lacks evidence of gain‐of‐function properties and its association with cancer is uncertain.

The pathogenic recessive *BLM* splice site variant c.2824‐2A > T was found in heterozygous state in two males diagnosed with UM at ages 45 and 65, with Stage T3a, IIB, and Stage T4b, IIIB UM, respectively (carrier frequency, 2/270; 0.74%; 95% CI 0.090 to 2.65, Table 3, Figure S2). The older patient with a more advanced melanoma died of metastases after less than 3 years whereas the other one is free of metastases after almost 9 years, neither had nonocular cancers. The overall allele frequency of c.2824‐2A > T in gnomAD v.2.1.1 is 1.1 × 10^−5^, and 5.6 × 10^−6^ in v.4.1.0 with three Finnish carriers, corresponding to 1 in 10,674 Finns (0.0094%, 95% CI 1 in 5,000 to 1 in 19). In our cohort, carriers of pathogenic germline *BLM* variants were more prevalent compared to individuals with pLOF *BLM* variants in the general Finnish population according to gnomAD v.4.1.0 (*p* = 0.024; odds ratio 9, 95% CI 1–35, Fisher's exact test, Table 3). Pathogenic germline *BLM* variants are associated with recessive Bloom syndrome, characterized by shorter stature, narrow face, skin rash, and early‐onset of various cancers, notably cutaneous squamous cell carcinoma, leukemia, lymphoma, and gastrointestinal tract cancer (Cunniff, Bassetti, and Ellis 2017).

Additionally, *BLM* c.2489C > T p.(Thr830Met) VUS was identified in a female with T1a, Stage I, UM at the age of 53 years and breast cancer at the age of 62 years (Table 2, Figure S2). Neither one has metastasized. The overall allele frequency of the VUS is 8.0 × 10^−6^ in gnomAD v.2.1.0 and 4.3 × 10^−6^ in v.4.1.0, with no Finnish carriers.

Following a comprehensive germline analysis of 19 genes linked to UM tumorigenesis, BAP1, or RCC in this large consecutive series of Finnish patients with UM, and considering our prior research on *BAP1* and *MBD4* in similar cohorts (Repo et al. 2020, 2019), the present findings affirm *BAP1* as the primary germline contributor to predisposition to UM in Finland. Specifically, pathogenic germline *BAP1* variants were present in 2% of unselected patients and 75% of those with FUM (Repo et al. 2019; Turunen et al. 2016), whereas no deleterious variants were detected in *MBD4* (Repo et al. 2020). Conversely, 0.4% of patients, as revealed by our study, harbor such variants in *BRCA1* or *MET* genes associated with dominant cancer risk. In one previous study, the frequency of *BRCA1* variants in UM (1/156, 0.64%) was nearer to that of the general population (0.26%) (Abdel‐Rahman et al. 2020) than in the Finnish population (0.37% vs. 0.056%), while the association of the *MET* variant detected with cancer remains uncertain.

To the best of our knowledge, two patients with UM have previously been identified with a pathogenic germline *BRCA1* variant. The first one, c.2603C > G, p.(Ser868*), was described in a proband with UM and prostate cancer, along with a family history of lymphoma, breast, stomach, cervical, skin, and liver cancers (Abdel‐Rahman et al. 2020). The second, a deletion spanning exon 20, was identified in a female with UM at 42 years of age, followed by breast cancer, and with a familial history of breast cancer (Cruz et al. 2011). Previous studies have also implicated *BRCA2* (Cruz et al. 2011; Iscovich et al. 2002; Sinilnikova et al. 1999) and *FLCN (*Fontcuberta et al. 2011) in patients with UM. Neither one harbored pathogenic variants in our consecutive cohort of Finnish patients with UM.

Moreover, the coding regions of six genes associated with UM tumorigenesis (*GNAQ*, *GNA11*, *CYSLTR2*, *PLCB4*, *EIF1AX*, or *SF3B1*) revealed no pathogenic germline variants and they are not expected to predispose to UM. This is consistent with previous studies, which found no pathogenic germline variants in *GNA11* or *GNAQ* in patients with UM (Abdel‐Rahman et al. 2011; Hawkes et al. 2013; Onken et al. 2008). The hotspot variations R183 and Q209 characteristic of UM are speculated to be incompatible with life (Piaggio et al. 2019), supported by observing them only in somatic or mosaic state (Van Trigt, Kelly, and Hughes 2022). Similarly, the absence of reports of *CYSLTR2*, *PLCB4*, *SF3B1*, or *EIF1AX* driver variants appearing in the germline, despite tumor analysis utilizing corresponding germline DNA, suggests that such variants are not identified in patients with UM elsewhere either (Field et al. 2018; Johansson et al. 2016; Nell et al. 2021).

BLM is a DNA helicase and a “caretaker” tumor suppressor, maintaining genomic integrity and preventing DNA damage accumulation (Hickson 2003). While carriers of pathogenic germline *BLM* variants do not exhibit a clear cancer risk, the variants likely increase cancer susceptibility when combined with other risk factors. For instance, pathogenic *BLM* variant carriers have lower tolerance to asbestos, leading to increased incidence of mesothelioma, and *BLM* loss is associated with higher frequency of intestinal tumors in Apc^Min^ mice (Bononi et al. 2020; Cunniff, Bassetti, and Ellis 2017; Goss et al. 2002; Hickson 2003). Thus, *BLM* variants might not predispose to UM but could amplify risk in the presence of coinciding risk factors, whether genetic or environmental. Given that no consistent environmental factors and only a few risk loci in addition to the genes already mentioned have been identified for UM (Mobuchon et al. 2017, 2022; Thomsen et al. 2020), it is likely that as yet unidentified risk factors contribute to the variable incidence of UM by ethnicity and latitude (Aronow, Topham, and Singh 2018; Kivelä 2014) and its familial occurrence (Abdel‐Rahman et al. 2020; Rai et al. 2017; Repo et al. 2019).

Our study has inherent limitations. Without primary tumor biopsies, the causative relationship between each germline variant and UM remains unconfirmed, leaving the possibility of chance association. Additionally, the short‐read sequencing might not detect large rearrangements or copy‐number variations, and the 30% VAF cutoff could make low frequency variants to go undetected.

To conclude, other UM drivers than *BAP1* do not harbor pathogenic germline variants in Finnish patients with UM and are not expected to predispose to UM. Our germline analysis of genes associated with the BAP1 protein did not explain familial UM predisposition. Hence, other genetic factors contributing to UM likely still need to be discovered.

## Ethics Statement

A written informed consent was obtained from all participants. The project was approved by the institutional review board of the Hospital Region of Helsinki and Uusimaa on June 11, 2012, and it followed the tenets of the Declaration of Helsinki.

## Conflicts of Interest

JAT received lecture fees from Thea Finland and Santen Finland, served in the advisory board of Novartis Finland, and as a consultant for Maculaser Oy and Pixieray Oy. TTK received lecture fees from Santen Finland. All are unrelated to this work. All other authors did not report a conflict of interest.

## Supporting information


**Figure S1.** A List of the 19 genes analyzed and the inclusion criteria. *BAP1* and *MBD4* are included for comparison but not presently analyzed. **B** Export from the String database showing the interrelations of the analyzed genes.

**Figure S2.** Representative Integrative Genomics Viewer (IGV) captures of targeted amplicon sequencing reads show identified variants of pathogenic, likely pathogenic and unknown significance. Variant allele frequency (VAF) was calculated from the total sequencing depth (DP) observed in IGV.

## Data Availability

The data that support the findings of this study are available from the corresponding author upon reasonable request.
