# Supplementary material for: Pathogenic Germline Variants in Uveal Melanoma Driver and BAP1‐Associated Genes in Finnish Patients with Uveal Melanoma
Source: Pigment Cell Melanoma Res. 2024 Sep 30;38(1):e13198. doi: 10.1111/pcmr.13198 (PMC11681845; doi:10.1111/pcmr.13198)

Supporting Information Figure S1

A

Gene	UM tumorigenesis driver	BAP1 interaction	In germline of patients with UM	Phenotype association
ASXL1 (NM_015338.6)	-	X	-	*de novo*/somatic mosaic, AD Bohring-Opitz syndrome (OMIM 605039, association with cancer risk pending)
ASXL2 (NM_018263.6)	-	X	-	*de novo*, AD Shashi-Pena syndrome (OMIM 617190)
BLM (NM_000057.4)	-	-	-	AR Bloom syndrome (OMIM 210900, **all types of cancer**)
BRCA1 (NM_007294.4)	-	X	X	AD Breast & Ovarian Cancer (OMIM 604370) & possibly others including **RCC**
BRCA2 (NM_000059.4)	-	-	X	AD Breast & Ovarian Cancer (OMIM 612555) & possibly others including **RCC**
*BAP1**	X		X	AD Tumor predisposition syndrome (OMIM 614327, uveal melanoma, cutaneous melanoma, malignant mesothelioma, clear cell renal cell carcinoma and several others)<br>*de novo* AD Kury-Isidor syndrome (OMIM 619762)
*MBD4**	X		X	AR Tumor Predisposition Syndrome (OMIM 619975, acute myeloid leukemia, myelodysplastic syndrome, colorectal adenomatous polyposis and carcinoma, and uveal melanoma + others)<br>AD, proposed risk of UM (OMIM 606660)
CYSLTR2 (NM_001308476.3)	X	-	-	-
EIF1AX (NM_001412.4)	X	-	-	-
FLCN (NM_144997.7)	-	-	X	AD Birt-Hogg-Dube syndrome (OMIM 135150, fibrofolliculomas, lung cysts, **RCC**)
FOXK2 (NM_004514.4)	-	X	-	-
GNA11 (NM_002067.5)	X	-	-	AD Hypocalcemia (OMIM 615361)<br>AD Hypercalcemia (OMIM 145981)
GNAQ (NM_002072.5)	X	-	-	somatic mosaic, AD Sturge-Weber syndrome (OMIM 185300)
HCFC1 (NM_005334.3)	-	X	-	XL, X-linked intellectual disability with methylmalonic acidemia and hyperhomocysteinemia (OMIM 309541)
KDM1B (NM_001364614.2)	-	X	-	-
MET (NM_000245.4)	-	-	-	AD Papillary **RCC** (OMIM 605074)<br>AR Deafness (OMIM 616705), association pending confirmation
PLCB4 (NM_001377142.1)	X	-	-	AR Auriculocondylar syndrome (OMIM 620458)<br>AD Auriculocondylar syndrome (OMIM 614669)
RBBP7 (NM_002893.4)	-	X	-	-
SETD2 (NM_014159.7)	-	-	-	*de novo* AD Luscan-Lumish syndrome (OMIM 616831)<br>*de novo* AD Rabin-Pappas syndrome (OMIM 620155)<br>Frequently somatically altered in **RCC**
SF3B1 (NM_012433.4)	X	-	-	-
VHL (NM_000551.4)	-	-	-	AD von Hippel-Lindau disease (OMIM 193300, retinal hemangiomas, cerebellar and spinal hemangioblastomas, **RCC**, pheochromocytoma + others)

**BAP1* and *MBD4* were not analyzed in this study

AD = autosomal dominant; AR = autosomal recessive; BC = breast cancer; RCC = renal cell carcinoma; UM = uveal melanoma

B

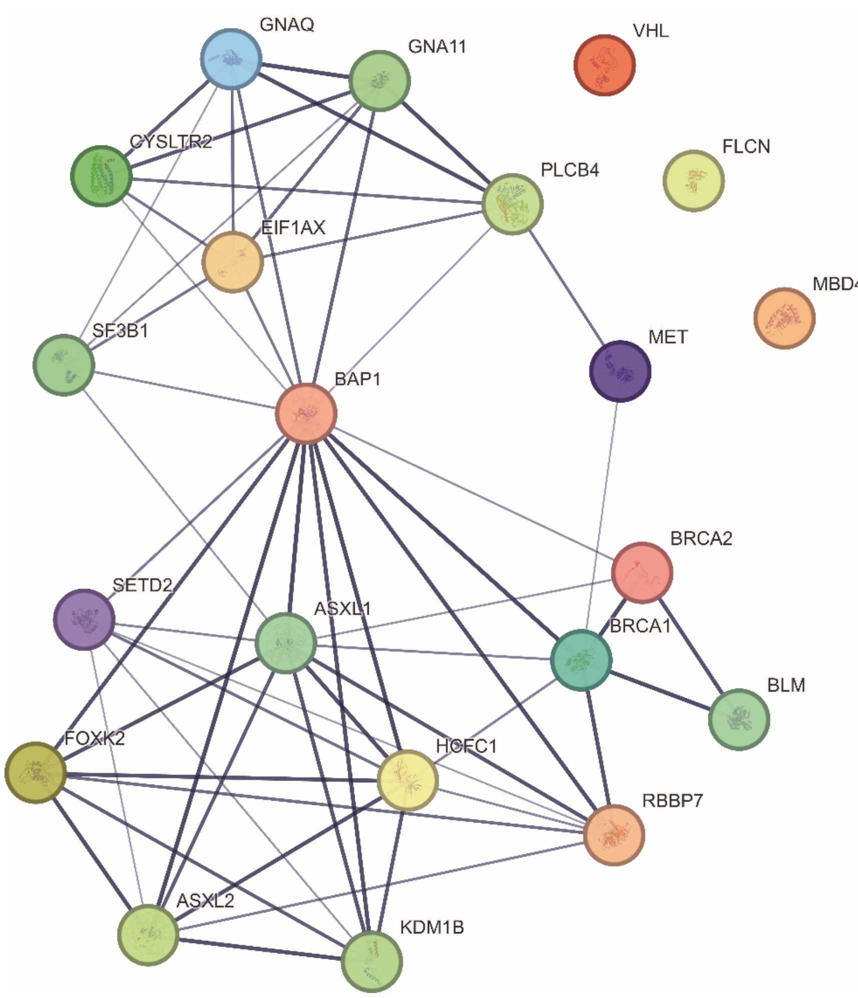

Supplement: Supplementary file 1 — Figure S1. A List of the 19 genes analyzed and the inclusion criteria. BAP1 and MBD4 are included for comparison but not presently analyzed. B Export from the String database showing the interrelations of the analyzed genes. [file PCMR-38-0-s001.pdf]
