# Supplementary material for: Pathogenic Germline Variants in Uveal Melanoma Driver and BAP1‐Associated Genes in Finnish Patients with Uveal Melanoma
Source: Pigment Cell Melanoma Res. 2024 Sep 30;38(1):e13198. doi: 10.1111/pcmr.13198 (PMC11681845; doi:10.1111/pcmr.13198)

Supporting Information Figure S2

UMG11-37 *BRCA1* c.3626del Leu1209*
(VAF 53%, DP 361)

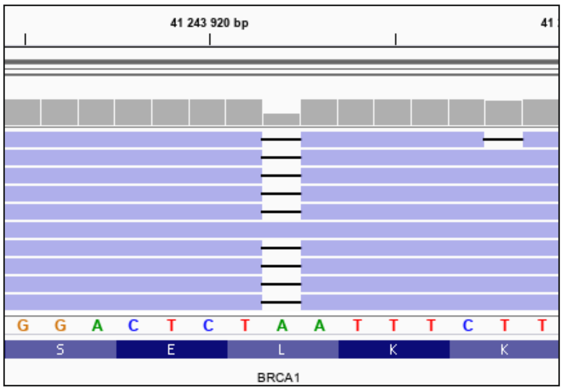

UMG14-11 *MET* c.252C>G Tyr84*
(VAF 66%, DP 106)

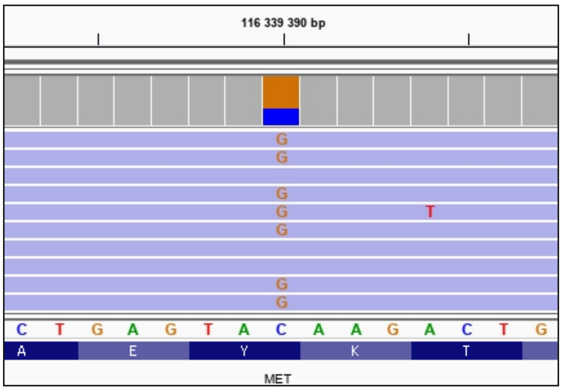

UMG14-24 *BLM* c.2824-2A>T
(VAF 51%, DP 43)

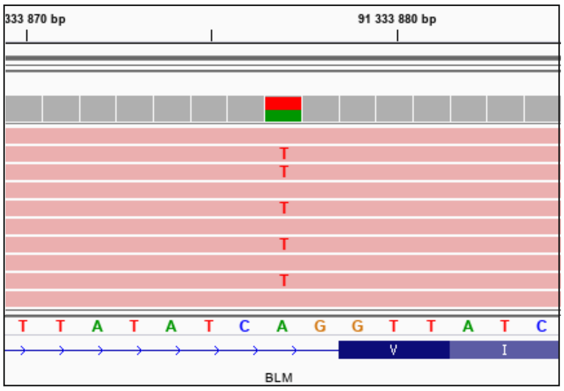

UMG14-52 *BLM* c.2824-2A>T
(VAF 44%, DP 119)

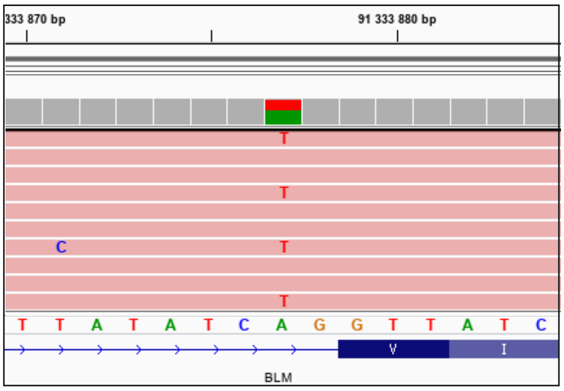

UMG13-38 *BLM* c.2489C>T Thr830Met
(VAF 57%, DP 65)

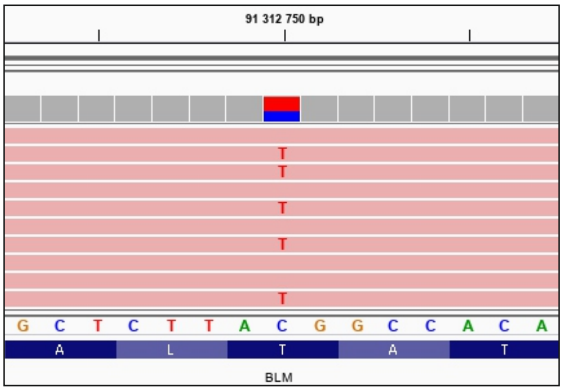

Supplement: Supplementary file 2 — Figure S2. Representative Integrative Genomics Viewer (IGV) captures of targeted amplicon sequencing reads show identified variants of pathogenic, likely pathogenic and unknown significance. Variant allele frequency (VAF) was calculated from the total sequencing depth (DP) observed in IGV. [file PCMR-38-0-s002.pdf]
